# Task-Independent Cognitive Workload Discrimination Based on EEG with Stacked Graph Attention Convolutional Networks

**DOI:** 10.3390/s25082390

**Published:** 2025-04-09

**Authors:** Chenyu Wei, Xuewen Zhao, Yu Song, Yi Liu

**Affiliations:** 1 Tianjin Key Laboratory for Control Theory and Applications in Complicated Systems, School of Electrical Engineering and Automation, Tianjin University of Technology, Tianjin 300384, China; wei@stud.tjut.edu.cn (C.W.); sy@email.tjut.edu.cn (Y.S.); 2 College of Mechanical and Electrical Engineering, Harbin Engineering University, Harbin 150001, China

**Keywords:** electroencephalogram, cognitive workload, functional connect, graph neural network, graph theory

## Abstract

In the field of neuroeconomics, the assessment of cognitive workload is a crucial issue with significant implications for real-world applications. Previous research has made progress in task-based germane cognitive load classification, but decentralized studies focusing on task-independent assessment have often produced less than optimal results. In this study, we present a stacked graph attention convolutional networks (SGATCNs) model to tackle the challenges related to task-independent cognitive workload assessment using EEG spatial information. The model employs the differential entropy (DE) and power spectral density (PSD) features of each EEG channel across four frequency bands (delta, theta, alpha, and beta) as node information. For the construction of the network structure, phase-locked values (PLVs), phase-lag indices (PLIs), Pearson correlation coefficients (PCCs), and mutual information (MI) are utilized and evaluated to generate a functional brain network. Specifically, the model aggregates spatial information on the dynamic map by stacking the graph attention layers and utilizes the convolution module to extract the frequency domain information from between the networks under each frequency band. We conducted a cognitive workload experiment with 15 subjects and selected three representative psychological experimental task paradigms (N-back, mental arithmetic, and Sternberg) to induce different levels of cognitive workload (low, medium, and high). Our framework achieved an average accuracy of 65.11% in recognizing the task-independent cognitive workload across the three scenarios.

## 1. Introduction

Cognitive load refers to the total amount of limited cognitive resources consumed by an individual during the execution of a task due to the application of specific strategies (e.g., “means-end analysis”), reflecting the task’s demand on the cognitive system and characterizing the occupation rate of mental resources under the given working conditions [1]. In cognitive psychology, the cognitive workload is used to describe the pressure or burden that an individual experiences during cognitive activities such as learning, problem solving, and performing tasks. Therefore, appropriate workload levels are required to achieve high efficiency without the individual being overloaded. The common cognitive workload measurement methods can be systematically categorized into three types based on their applicability in perceptually rich environments: performance measurement, subjective measurement, and physiological measurement [2]. Among them, researchers have recently used physiological signals to identify the cognitive workload status of individuals, including electroencephalograms (EEGs) [3], functional magnetic resonance imaging (fMRI) [4], electrocardiograms (ECGs) [5], and the galvanic skin response (GSR) [6]. The cognitive workload is closely related to the higher cognitive functions of the brain, including working memory, in which the frontal region of the cerebral cortex plays an important role.

More and more researchers have become devoted to exploring the relationship between high temporal resolution EEGs and subjects’ mental states in cognitive workload recognition. Mahesh et al. [7] utilized a multisampling-rate infinite-impulse-response filter bank to decompose EEG signals into 10 frequency bands, and extracted multiple features from them for cognitive workload classification using integrated subspace KNN classifiers. This study demonstrated that decomposing EEG signals into multiple frequency bands can enhance the recognition accuracy of workload classification. Additionally, Yang et al. [8] extracted temporal features and PSD features from EEG signals, proposing the ensemble stacked denoising autoencoder classifier with local information preservation (EL-SDAE) to improve the accuracy of cognitive workload recognition. The above studies considered the time or frequency features of EEG signals, but lacked exploration of the spatial domain information of EEG signals.

Neuroscience research has demonstrated that the brain’s functions are not carried out independently by single brain regions, but require intricate interactions among multiple brain regions [9]. Understanding how to leverage the spatial information of these different brain regions to enhance cognitive workload recognition would provide potential solutions for task-independent cognitive workload discrimination. Wang et al. [10] used an attention-based recursive fuzzy network to explore the association between EEG features and workload. Their results demonstrated that imposing adaptive attention weights on dynamic connections significantly improved the classification accuracy across N-back and mental arithmetic tasks. Kwak et al. [11] extracted spatial and spectral information from a 3D convolutional layer, and fused the multi-level features extracted from the convolutional layer by using weighting factors, enhancing the performance of a CNN in workload estimation. Ramaswamy et al. [12] proposed a spatio-temporal EEG network (EEG-TopoNet) which used the PSD and entropy to generate brain terrain as inputs to extract spatial features and capture brain activation patterns at different durations, and the results showed that the brain terrain map network based on PSD features achieved the best classification results. However, most of the above studies are based on single tasks, and the exploration of cognitive workload recognition in task-independent scenarios is limited.

Taking all the above into consideration, we propose a stacked graph attention convolutional networks (SGATCNs) model to achieve cross-task cognitive workload recognition. Firstly, to collect cognitive workload EEG datasets under different tasks, we designed three tasks using NASATLX as the evaluation criterion, with each task containing three levels of cognitive workload. Secondly, multiple functional connectivity matrices were collected and a functional brain network was constructed, with frequency domain features as the information features of the nodes so that discrete graph data could be constructed as input to the model. In the SGATCNs model, the SGAT structure is used to learn spatial EEG representations in different frequency domains, where the graph attention mechanism can assign higher weights to key nodes in adjacent nodes to achieve more efficient spatial information aggregation and updating. Subsequently, convolutional layers are used to extract frequency-domain information from the features in one step, thereby obtaining high-dimensional feature data to compensate for the insufficient learning ability of graph neural networks regarding frequency-domain information.

The main research content of each segment is as follows: Section 2 focuses on the work related to cognitive workload recognition based on brain networks and deep learning. Section 3 describes the whole process of the signal acquisition experiment. Section 4 describes the feature extraction and classification model. Section 5 details the experimental results. Section 6 discusses this paper. Finally, Section 7 gives the conclusion.

## 2. Related Works

### 2.1. Functional Brain Network

A functional brain network is a kind of brain network constructed on the basis of functional connectivity features which is capable of describing the structure between different regions of the brain as well as the functional connectivity patterns between them on a macroscopic scale [13]. Nafise et al. [14] identified patterns of functional brain connectivity, revealing that the frontal and parietal lobes tend to aggregate more under low workload than under high workload. Guan et al. [15] constructed a dynamic functional connectivity network under different microstates by calculating the phase-locked values between EEG channels on the basis of microstates analysis, and identified the cognitive workloads across various tasks with a support vector machine classifier time. Dimitrakopoulos et al. [16] applied phase lag index features to construct a functional brain network, and calculated network properties such as the global clustering coefficients, characteristic path lengths, and small-world indicators of the network as features to identify the cognitive workload based on graph-theoretic methods. The above studies show that the study of functional brain network structures can effectively characterize the interaction of information between different brain regions during changes in cognitive workload.

We constructed a functional brain network structure using four correlation methods and extracted PSD and DE features as node information, sparsified the functional brain network with different sparsity ratios, and searched for the functional brain network structure that best characterizes the degree of cognitive workload through deep learning models.

### 2.2. Classification Model Based on Cognitive Workload

In contrast to classical machine learning algorithms, deep learning methods can learn complex information from multiple dimensions simultaneously. Ji et al. [17] proposed a dynamic residual network with attention mechanism (DRNA-Net) which is able to find more stable EEG features in different cognitive tasks by combining a recurrent network (DRNA-Net) and a dynamic residual network with attention mechanism (DRNA-Net), which allows it to find more stable EEG features in different cognitive tasks by combining the recurrent network with the self-attention mechanism. Zhou et al. [18] proposed to construct a cross-task cognitive workload recognition model based on EEG, which improves the cross-task recognition accuracy using a domain adaptive approach. Gupta et al. [19] proposed a cognitive workload estimation method based on EEGs, the functional brain connectivity, and deep learning which can classify the cognitive workload in near real-time by extracting model-free functional connectivity metrics and applying deep learning architectures (CNN, LSTM, and Conv-LSTM).

However, traditional deep learning methods are mainly used with Euclidean data, and in the face of the brain network structure, graph neural networks can be more effective in topologizing the information between nodes. Zhang et al. [20] proposed a graph convolutional network based on the compression and excitation (SE) block, and transformed the resulting dimensionality-reduced EEG signals into graph data based on the Pearson’s correlation coefficient to achieve the identification of the cognitive workload. However, the existing studies based on graph neural networks ignore the importance of frequency domain features; therefore, we construct functional brain network structures under different frequency bands and use them as inputs to a SGATCNs model in the form of dynamic graphs, and fully extract the frequency domain information using convolutional layers.

## 3. Materials

### 3.1. Subjects

Fifteen graduate students from Tianjin Polytechnic University were recruited for this experiment. Eleven of them were male and four were female, and the mean age of the subjects was 24 years old (aged between 22 and 25 years old). All subjects were native Chinese speakers, right-handed, and had normal or corrected-to-normal vision. After the experiment was explained to the subjects, the subjects signed an informed consent form. Meanwhile, this experiment was approved by the Ethics Committee of Tianjin University of Technology. Application No. TUT20221705 was obtained in 12 May 2022.

### 3.2. Experimental Setup

Three types of psychological experimental paradigms, N-back, mental arithmetic (MA), and Sternberg, were selected as the target tasks for the experiments, and each experiment consisted of three levels (low workload task, medium workload task, and high workload task). The tasks were realized by Psychtoolbox (PTB). During the experiment, subjects were asked to remain relaxed, sit still in front of the computer, and focus on the cross in the middle of the screen as much as possible; they were also asked to avoid frequent blinking during the experiment.

**Figure 1:**
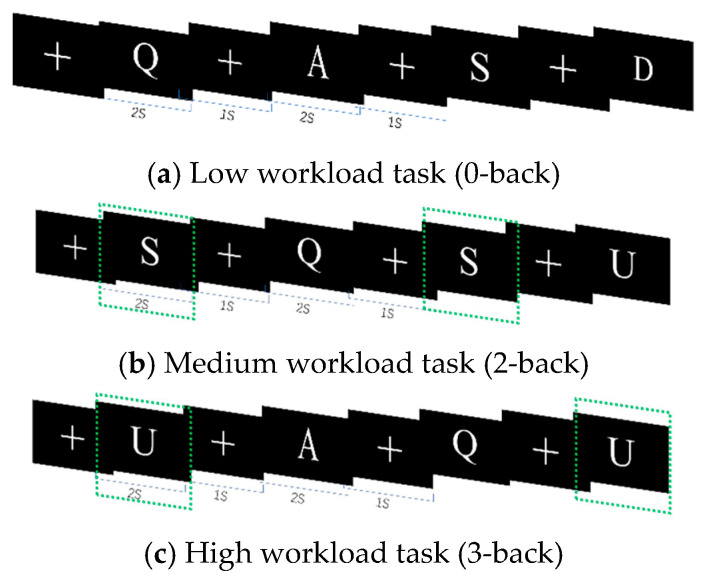
N-back experiment.

The N-back experiments included a working memory task with three levels of difficulty: 0-back workload, 2-back workload, and 3-back workload. During the experiment, subjects would see random letters on the monitor as test stimuli; the presentation time was 1 s, and there was a 2 s interval between every two test stimuli. The experimental paradigm is shown in Figure 1. In the 0-back task, the letter “A” was used as the stimulus target, and subjects were asked to judge whether the current letter was the stimulus target. In the 2-back task, the second previous letter was used as the stimulus target in each of the subjects’ judgments. In the 3-back task, the third previous letter would be considered the stimulus target. Subjects were asked to determine whether the current letter was the same as the stimulus target, and if so, to press the “F” key on the keyboard. Otherwise, they were instructed to press the “J” key.

**Figure 2:**
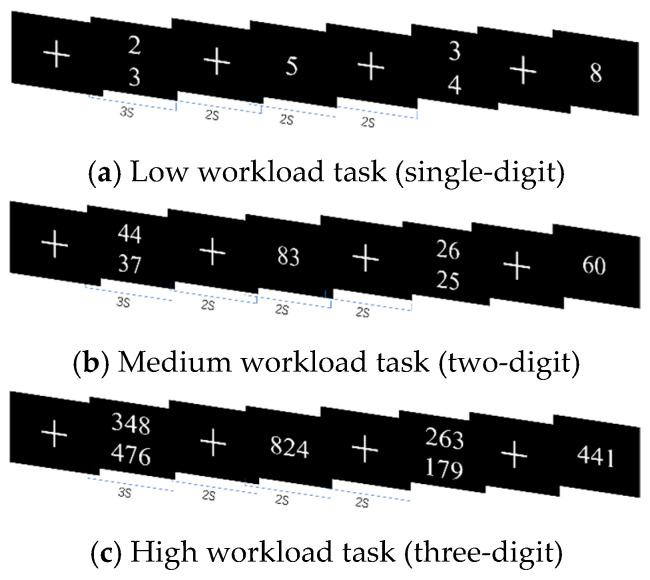
Mental arithmetic experiment.

In the mental arithmetic (MA) experiments, as the cognitive workload moved from low to high, the stimulus targets were addition operations with single-digit, two-digit, and three-digit numbers, as shown in Figure 2. During the experiment, the subject would see two numbers, each for a duration of 3 s, and after a 2-s interval, a number consistent with or similar to the correct answer would be displayed on the computer screen for 2 s. At this point, the subject was asked to judge whether the results displayed were correct. If so, they were instructed to press the keyboard “F” key, and otherwise to press the “J” key.

**Figure 3:**
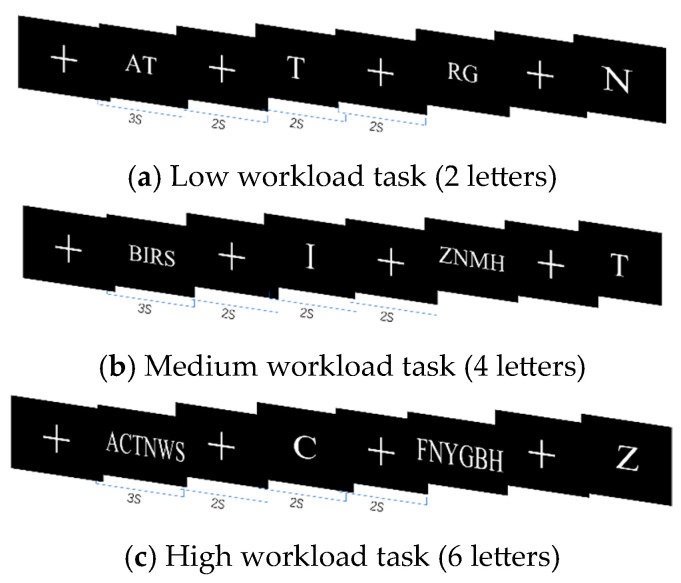
Sternberg experiment.

Sternberg experiments achieve different task workloads by controlling the number of letters: low workload tasks include 2 letters, medium workload tasks include 4 letters, and high workload tasks include 6 letters. During the experiment, memory items and target items would appear on the screen in sequence, as shown in Figure 3. The subjects needed to remember the letters appearing in the memory item, the duration of was 3 s. The target item would appear on the screen after a delay of about 2 s and, at this time, the subjects would need to press a key to react to whether the current letter appeared in the memory item. If yes, they were instructed to press the “F” key on the keyboard and otherwise to press the “J” key. Then, the answer would be displayed for 2 s.

**Figure 4:**
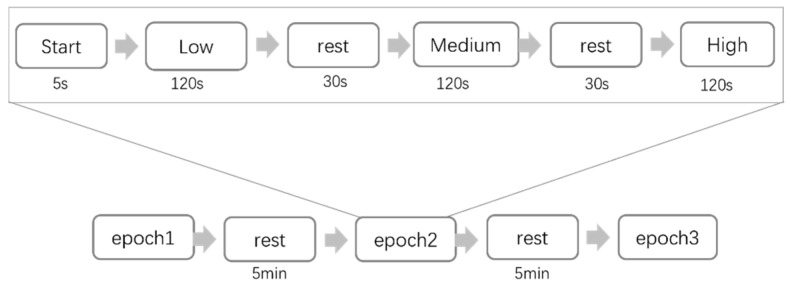
Cognitive workload elicitation program.

Before the experiment, the subjects were instructed to read the experimental procedure, and a pre-experiment was conducted to assist the subjects in familiarizing themselves with the experimental process and requirements. As depicted in Figure 4, each subject undertook a total of three rounds of experiments, with each round comprising three groups of tasks and each group of tasks encompassing three workload levels: low, medium, and high. Each level corresponded to an experiment duration of approximately 2 min, and these were accompanied by a 30-s break between experiments of different workload levels, a 5-min break for the subjects between each group of tasks, and a 15-min interval between each round of experiments. The subjects had ample time to rest and recover, which minimized the potential influence of fatigue on the experimental results. The EEG signals were collected throughout the whole procedure, and for their collection we used the NeuSen.W64 system, with a sampling frequency of 1000 Hz and in which the electrode arrangement of the 59 EEG channels conformed to the international 10–20 system standard. The Ethics Committee of Tianjin University of Technology approved the experiment.

### 3.3. Feasibility Analysis via the NASA-TLX Workload Scale

**Figure 5:**
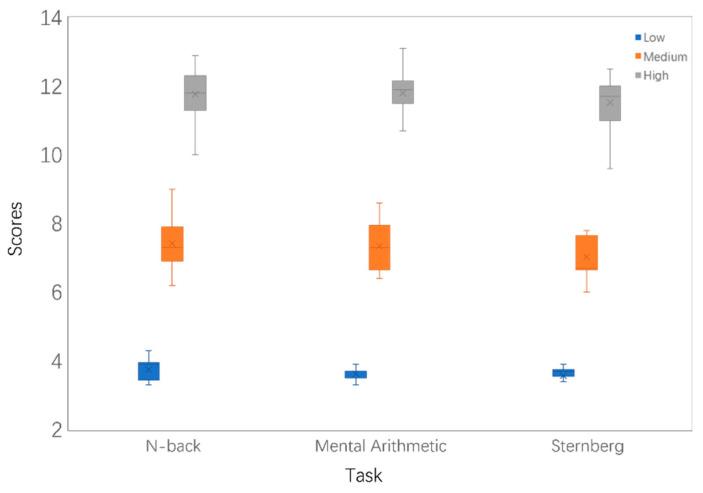
The NASA-TLX scores of 15 participants on three tasks with different cognitive workload levels.

In order to verify the correlations of the proposed three types of paradigms with different workload levels, 15 subjects completed each trial and then the three workload levels under different paradigms were scored using the NASA-TLX scale, the results of which are shown in Figure 5. The NASA-TLX scores under each workload level for the three tasks differed significantly, and the mean values of the scores for the three tasks under different workloads were 3.64, 7.26, and 11.64, respectively. Comparing the results of the NASA-TLX scores of the three tasks under different workloads, the differences in scores between the different tasks at the same level of difficulty were small and the differences in scores between the different levels of difficulty were large.

## 4. Methods

**Figure 6:**
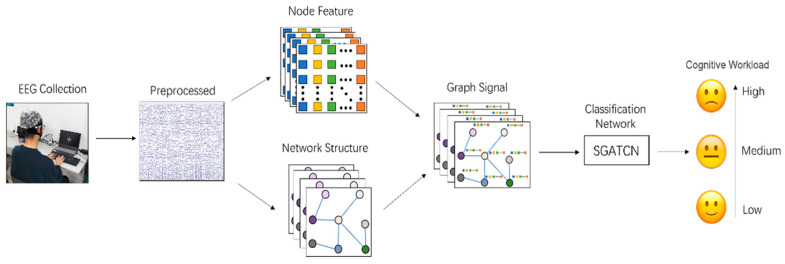
The cognitive workload recognition process based on EEG signal with SGATCNs.

The model framework is shown in Figure 6. The first step was to preprocess the raw EEG signals, and then extract the functional brain network construction and node information composed of graph signals, respectively, which was followed by the use of the SGATCNs classifier to identify different levels of cognitive workload.

### 4.1. EEG Preprocessing

The raw EEG signals were preprocessed using the EEGLAB toolbox. First, the sampling frequency was down-sampled from 1000 Hz to 200 Hz. Filtering operations were performed on the EEG signals by bandpass filters (1–75 Hz) and trap filters (49–51 Hz) to eliminate the high and low frequency noise and IF interference. A global averaging reference was applied for re-referencing to enhance the signal. The bad channels were corrected by removing the bad channel, while the original signals were decomposed by independent component analysis (ICA) and the artifactual components such as the ophthalmic, oculomotor, EMG, and ECG values were removed from the original signals to obtain relatively pure EEG data. The EEG samples obtained under each task were divided by a time window of 3 s. A total of 1080 EEG samples were collected from each subject, and the EEG signals were categorized into four frequency bands, delta (1–3 Hz), theta (4–8 Hz), alpha (8–12 Hz), and beta (12–30 Hz) using the Butterworth filter.

### 4.2. Node Information Extraction

EEG signals are represented as continuous time series, and information related to cognitive workload can be extracted from them by analyzing their frequency domain features. Common frequency domain features in cognitive workload recognition include the power spectral density (PSD), differential entropy (DE), etc. PSD usually represents the variation in signal power with the frequency. Fast Fourier transform (FFT) was used to extract the PSD features in each frequency band of the non-overlapping Hamming window [21]. The average power of the EEG electrode channel in the time period $t\in \left[-\frac{T}{2},\frac{T}{2}\right]$ can be expressed as follows:

(1) $$P=\frac{1}{T}\int_{-\frac{T}{2}}^{\frac{T}{2}}f^{2}\left(t\right)dt$$

The Fourier transformation of f(t) for time period $t\in \left[-\frac{T}{2},\frac{T}{2}\right]$ is $f\left(\omega \right)=F_{T}[f(t)]$. F[·] denotes the Fourier transformation. When T→∞, $\frac{{\left|F_{T}\left(\omega \right)\right|}^{2}}{2\pi T}$ will converge to some limit value, then the power spectral density in the frequency domain can be expressed as follows:

(2) $$P=\underset{T\to \infty }{lim}\frac{1}{T}\int_{-\frac{T}{2}}^{\frac{T}{2}}f^{2}\left(t\right)dt=\frac{1}{2\pi }\int_{-\infty }^{\infty }\underset{T\to \infty }{lim}\frac{{\left|F_{T}\left(\omega \right)\right|}^{2}}{T}d\omega$$

DE usually describes the amount of uncertainty variation in a continuous signal. The DE is defined as follows:

(3) $$\;DE\left(X\right)=\frac{1}{2}\log\left(2\pi e\sigma^{2}\right)$$

where the time series X obeys a Gaussian distribution $N(\mu ,\sigma^{2})$.

### 4.3. Functional Brain Network Construction

The key to constructing the graph structure is to determine the functional connectivity index of the brain network. In order to ensure that the functional connectivity indexes can well reflect the correlations between channels, this paper explores four different functional connectivity indexes to construct the collocation matrix with different levels of sparsity.

The phase locking value (PLV) measures the strength of synchronization between nodes on the scalp surface and is commonly used in studies of EEG signals to investigate task-induced changes in the long-range synchronization of neural activity. The PLV synchronization relationship between two nodes can be expressed as follows:

(4) $$PLV=\left|{\left\langle e^{i\phi_{xy}\left(t\right)}\right\rangle }_{t}\right|=\sqrt{{\left\langle \cos\phi_{xy}\left(t\right)\right\rangle }_{t}^{2}+{\left\langle \sin\phi_{xy}\left(t\right)\right\rangle }_{t}^{2}}$$

(5) $$\phi_{xy}\left(t\right)=\phi_{x}\left(t\right)-\phi_{y}\left(t\right)$$

where the pointed brackets represent the average time period, i represents the imaginary part, $\phi_{xy}$ is the instantaneous phase difference between the signals of the two nodes x(t) and y(t) at moment t, and $\phi_{x}\left(t\right)$ and $\phi_{y}\left(t\right)$ are the instantaneous phases at moment t, respectively.

The phase lag index (PLI) measures whether the relative phase distribution between nodes is asymmetric around 0 or π, and responds to the consistency of the signal of one electrode channel in relation to the signal of the other electrode channel by overshooting or lagging, which is determined by the following:

(6) $${\mathrm{PLI}}_{\mathrm{xy}}\left(f\right)=\left|<\mathrm{sign}\left(\phi_{x}\left(f\right)-\phi_{y}\left(f\right)\right)>\right|$$

where $\phi_{x}\left(f\right)-\phi_{y}\left(f\right)$ denotes the phase synchronization of the x-channel and y-channel signals at frequency f.

The Pearson correlation coefficient (PCC) is a linear correlation coefficient commonly used to reflect the degree of linear correlation between two variables, which is defined as the quotient of the covariance and standard deviation between two variables, as shown below:

(7) $$\rho_{XY}=\frac{Cov\left(X,Y\right)}{\sqrt{D\left(X\right)}\sqrt{D\left(Y\right)}}=\frac{E\left(\left(X-E\left(X\right)\left(Y-E\left(Y\right)\right)\right)\right)}{\sqrt{D\left(X\right)}\sqrt{D\left(Y\right)}}$$

where E(X) is the mathematical expectation, D is the variance, $\sqrt{D(X)}$ is the standard deviation, and Cov(X,Y) is the covariance.

Mutual information (MI) is a measure of interdependence between two random variables, denoted by I(X,Y). Mutual information measures the information shared by two random variables: the extent to which the uncertainty in the random variable Y is reduced when the random variable X is known:

(8) $$I\left(X,Y\right)=H\left(X\right)+H\left(Y\right)-H\left(X,Y\right)$$

where H(X,Y) represents the joint and entropy of variables X and Y.

For variable X, its information entropy can be expressed as:

(9) $$H\left(X\right)=-\sum_{i=1}^{n}p\left(x_{i}\right){log}_{2}\left[p\left(x_{i}\right)\right]$$

where $p(x_{i})$ is the probability function of variable X. Similarly, the joint and information entropy between X and Y can be expressed as:

(10) $$H\left(X,Y\right)=-\sum_{i=1}^{n}\sum_{j=1}^{m}p\left(x_{i},y_{j}\right){log}_{2}\left[p\left(x_{i},y_{j}\right)\right]$$

where $p\left(x_{i},y_{j}\right)$ is the joint probability of signals X and Y.

### 4.4. Network Topology Parameters

Complex network analysis methods based on graph theory provide powerful support for quantifying and analyzing the properties of functional brain networks, and the analysis of network topological parameters can effectively characterize the brain network state. We extracted 2 global parameters of the network: small-world properties and global efficiency, and 2 local parameters: node clustering coefficients and local efficiency, and analyzed the topological changes of the functional brain network under different workload levels.

The small-world property quantifies the closeness of the network, and a higher small-world property represents a relatively high energy efficiency of the network in the process of information transfer and processing. The small-world property is a measure of the small-world nature, which is defined as the ratio of the normalized clustering coefficient to the length of the normalized feature paths:

(11) $$\sigma =\frac{\frac{CC}{CC_{r}}}{\frac{L}{L_{r}}}$$

where the clustering coefficient CC of the network is the average of the clustering coefficients C of all nodes:

(12) $$CC=\frac{1}{N}\sum_{I=1}^{N}C_{i}$$

The shortest path length $L_{i,j}$ represents the minimum number of edges needed to connect node i to node j. The characteristic path length L represents the average shortest path length of the entire network [22], which is defined as the average of the shortest path lengths between all pairs of nodes:

(13) $$L=\frac{1}{N\left(N-1\right)}\sum_{i\neq j}L_{i,j}$$

where ${CC}_{r}$ and $L_{r}$ are the clustering coefficients and feature path lengths from random networks of the same size [23].

Global efficiency means that the efficiency of a path between any two vertices is defined as the reciprocal of the shortest distance between the vertices. When such a path does not exist, the global efficiency is zero [24]. The global efficiency is a measure of the overall efficiency of information processing transmission throughout the network, which is calculated as follows:

(14) $${\;E}_{glob}=\frac{1}{N\left(N-1\right)}\sum_{i\neq j}L_{i,j}$$

where $l_{ij}$ is the shortest path between nodes i and j is the minimum number of edges required to connect two nodes.

The node clustering coefficient C is an important measure of the degree of grouping of a network, which represents the likelihood that the neighboring nodes of a node i in the network can be neighboring nodes to each other [25]. The node clustering coefficient is defined as follows:

(15) $$C_{i}=\frac{2t_{i}}{k_{i}\left(k_{i}-1\right)}$$

where $C_{i}$ is the clustering coefficient of node i, $t_{i}$ is the number of triangles formed by node i, and $k_{i}$ is the degree of node i.

The local efficiency reflects the degree of differentiation among nodes in the network and also represents the local information transfer capability of the network and the ability of the network to defend against random attacks [26]. The local efficiency of any node can be expressed as:

(16) $$E_{loc}=\frac{1}{N_{G_{I}}(N_{G_{I}}-1)}\sum_{j\neq k\in G_{I}}\frac{1}{l_{jk}}$$

where $G_{I}$ is the subgraph consisting of the neighbors of the node. It measures the ability of a network node to propagate information to other nodes in the network.

### 4.5. Stacked Graph Attention Convolutional Networks (SGATCN)

**Figure 7:**
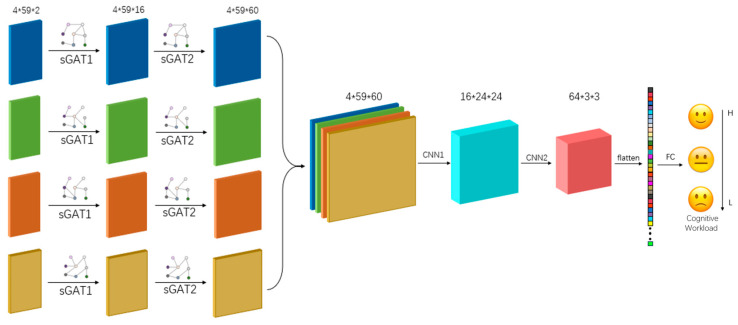
The structure of the SGATCNs classification model.

In this study, graph neural network and convolutional neural network structures are combined to learn EEG features from spatial and spectral dimensions. The proposed network consists of a stacked graph attention layer, a convolutional layer, and a fully connected layer, as shown in Figure 7. Traditional graph neural networks can only process static maps, and EEG signals in different frequency bands often contain different information, so the structure of the constructed brain network changes as the frequency domain changes. A single adjacency matrix cannot completely represent the brain network structure under different frequency bands, and the information aggregation of the graph structure under different frequency bands is performed by a stacked network model. The network takes a discrete graph, which is a kind of dynamic graph, as the input and represents the network structure through a series of discrete network snapshots:

(17) $$D_{G}=\left\{G_{1},G_{2},\ldots ,G_{N}\right\}$$

where N is the number of included graphs, $G_{N}$ is the Nth snapshot of the network, N=4, representing the four EEG bands, and the most singular graph structure can be represented as:

(18) $$G=\left(V,E\right)$$

where E is the adjacency matrix of the functional brain network and V is the set of node information.

The SGATCNs model applies two SGAT layers. The first SGAT layer receives the adjacency matrix of the discrete graph signals and the original node feature matrix to update its own node features against the aggregated neighboring node features of each node. The second SGAT layer receives the node features that contain information about each node’s own features and its first-order neighbors, and uses this as the basis for a further step of aggregation of node information.

The SGAT layer achieves information aggregation for each graph signal in the discrete graph signals by stacking the GAT structures, and the inputs for any GAT structure are $h=\left\{\vec{h_{1}},\vec{h_{2}},\vec{h_{3}},\ldots ,\vec{h_{N}}\right\},\vec{h_{i}}\in R^{F}$, where N is the number of nodes of the graph and F is the feature length of the input node information. For any two nodes i,j, the importance of the features of node j to node i can be expressed as the number of points and F is the feature length of the input node information. For any two nodes i, j, the importance of the features of node j to node i can be expressed as:

(19) $$e_{ij}=\alpha \left(W\vec{h_{i}}∥W\vec{h_{j}}\right)$$

For each node in the network, its $e_{ij}$, $j\in N_{i}$, $N_{i}$ represnts the neighbors of node i, and is calculated and normalized to obtain the attention coefficient of the network:

(20) $$a_{ij}=\frac{exp\left(LeakyRelu\left({\vec{a}}^{T}\left[W\vec{h_{i}}\;∥W\vec{h_{j}}\;\right]\right)\right)}{\sum_{k\in N_{i}}exp(LeakyRelu\left({\vec{a}}^{T}\left[W\vec{h_{i}}\;∥W\vec{h_{k}}\;\right]\right))}$$

The linear combination of corresponding features is computed by normalization coefficients, which completes the aggregation and updating of the information of the surrounding nodes and serves as the final output feature of that node:

The use of convolutional layers to process the output of the SGAT, which is conducted through multiple convolutional operations to extract the information between the frequency domains to obtain high-dimensional feature data and to complete the information transfer and coding of discrete map signals in the various frequency bands convolutional layer, is mainly implemented by sliding a convolution kernel on a feature matrix and through the carrying out convolution operation to obtain a new set of features. An image obtained after carrying convolutional operations to get the result is called a feature map. The convolutional layer can be described as:

(21) $$h(i,j)=(I*K)(i,j)=\sum_{m}\sum_{n}I(i-m,j-n)K(m,n)$$

where the input feature matrix is I, K is the convolution kernel, and $h\left(i,j\right)$ is the value of row i and column j of the output matrix.

The fully connected layer mainly handles the feature data output from the convolutional layer after tiling and then obtains the classification prediction results, which flattens the feature matrix output from the last graph’s convolutional layer into the fully connected layer and outputs the probability of each category through softmax after the nonlinear operation of the fully connected layer is conducted. The final prediction result of the model is the category with the largest output probability.

(22) $${\vec{h}}_{i}=\sigma \left(\sum_{j\epsilon N_{i}}\alpha_{ij}W\vec{h_{i}}\right)$$

## 5. Experimental Results

The model training was performed on a DELL (Fujian, China) G-15 laptop (CPU: i7-10200H, RAM: 16G, GPU: GTX 3060) while the proposed method was compared with two graph neural network classification methods, GAT [27] and GCN [28], and four machine learning algorithms, SVM [29], KNN [30], RF [31], and LR [32]. Table 1 shows the parameters of different classifiers.

### 5.1. Task-Independent Cognitive Workload Recognition via Subject-Independent Mapping

The task-independent cognitive workload recognition experiments were conducted by cross-validation, i.e., the dataset of one task was used as the test set and the datasets of the other two tasks were used as the training set, the three sets were crossed three times, and the average of the three experiments was used as the final result.

**Figure 8:**
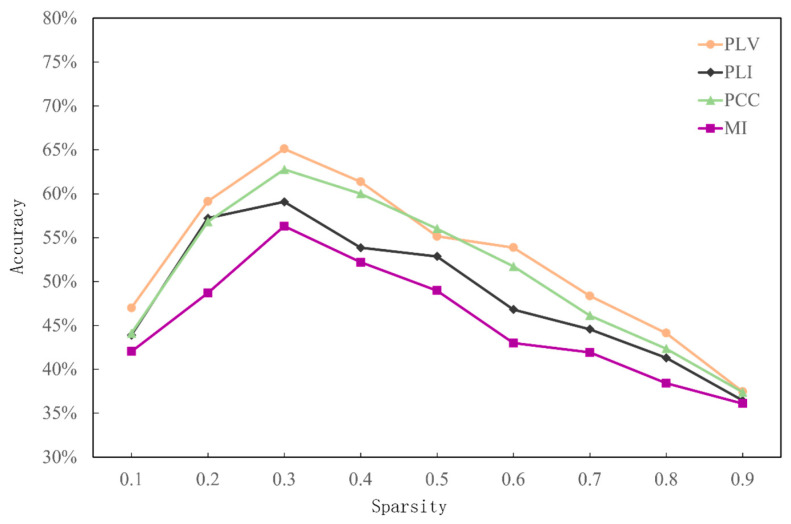
Effectiveness of task-independent cognitive workload categorization with different sparsities.

The task-independent cognitive workload recognition results of the four connectivity methods with different sparsities are shown in Table 2. The functional brain network structure based on PLV features has the highest recognition accuracy of 66.12% when using N-back as the test set. The average accuracy of the four connectivity methods with different sparsities is shown in Figure 8, and the highest classification accuracy was that based on PLV features and was up to 65.11%, while that based on MI features was only 56.31%. On the other hand, in all the connectivity methods, the model classification accuracy increased first and then decreased, and the average classification accuracy reached the highest in all of them when the sparsity decreased to 0.3.

Table 3 shows the recognition accuracies of the common algorithms when the PLV is selected with a sparsity of 0.3, and the results show that the SGATCNs model can obtain higher classification accuracy compared to other algorithmic models. The classification accuracy, precision, and F1 score of the SGATCNs model are higher than those of other classification models, as it obtained an accuracy of up to 65.11%, which is higher than those of the GAT and GCN models, which are the same as a graph neural network classifier. The experimental results show that the SGATCNs model is able to integrate frequency domain features and spatial domain features in the form of graph data, and at the same time, it can effectively utilize the EEG inter-channel topology to learn more discriminative cognitive workload representations of EEG signals.

### 5.2. Task-Independent Cognitive Workload Recognition via Subject-Dependent Mapping

**Figure 9:**
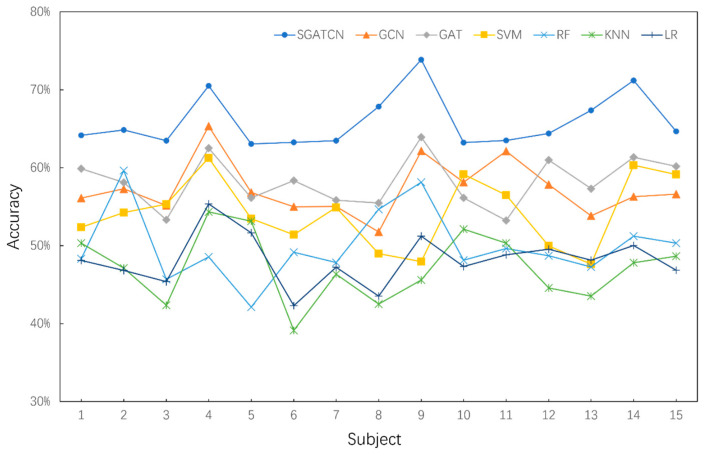
Classification accuracy of 15 subjects with different classifiers.

A brain network was constructed based on the PLV connection matrix at a sparsity of 0.3 for subject-dependent cross-task cognitive workload-based experiments, and Figure 9 shows the results of task-independent cognitive workload recognition for 15 subjects under seven classifiers. The proposed model achieved the highest classification accuracy for each subject’s data, indicating that the model has high generalizability and high applicability of performance for different subjects. For all subject, the task-independent classification accuracies exceeded 60%, with six subjects having accuracies of over 65% and three subjects having accuracies of over 70%. Subject 9 had the highest accuracy of 73.86%, while subject 5 had the lowest average accuracy of 63.05%.

**Figure 10:**
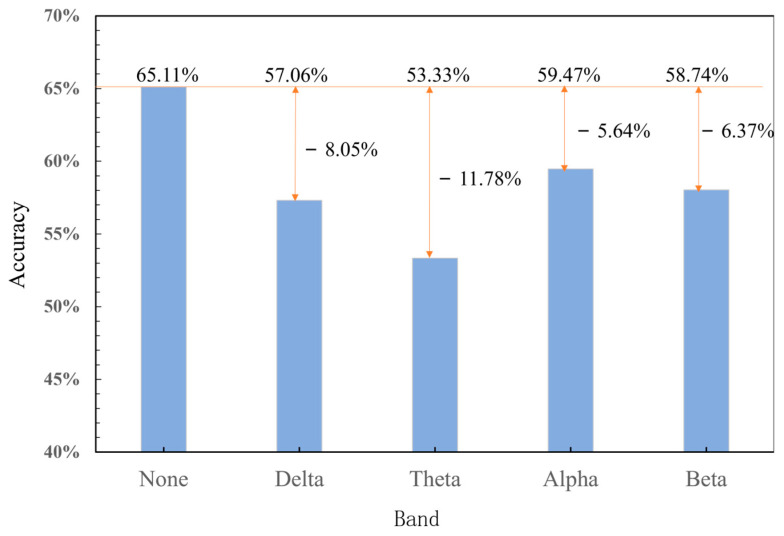
Classification after shielding different frequency bands.

To further explore the classification accuracy under different frequency bands, Figure 10 shows the task-independent cognitive workload recognition results of the model after one of the frequency bands was masked. In the experiment, the PLV was used as the basis for constructing the functional brain network, and the network sparsity was set to 0.3. The results showed that the classification accuracy of the model after each frequency band was shielded showed different degrees of decline, among which shielding out the theta band had the greatest impact on the accuracy with a recognition accuracy of 53.33%, which was a decrease of 11.78%. This was followed by the alpha band, which exhibited a decrease of 8.05%. the delta band and beta band had relatively lower impacts, decreasing by 5.64% and 6.37%, respectively, i.e., the theta band has a better feature characterization ability in cross-task cognitive workload recognition based on the SGATCNs model.

## 6. Discussion

### 6.1. Network Global Parameter Analysis

In order to analyze the network topology structure during the changes in cognitive workload, the significance of each parameter was tested through variance analysis and statistical analysis was performed. First, a single-factor variance analysis was performed on the small-world attributes and global efficiency of the functional brain network under the three workload levels to obtain the corresponding statistic F and test level *p*. The analysis results are shown in Table 4.

**Figure 11:**
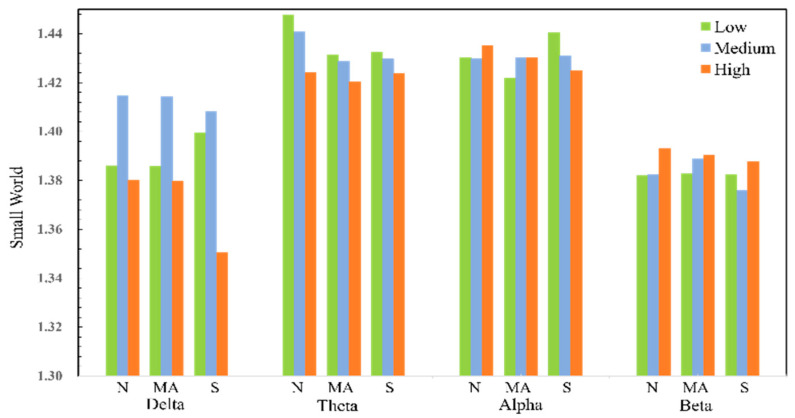
Statistical results of small-world properties in each frequency band.

**Figure 12:**
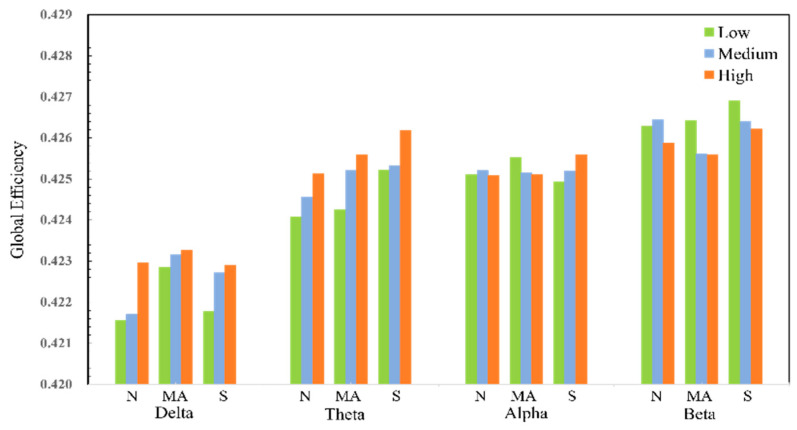
Global efficiency statistical results under each frequency band.

The data in the Table 4, at *p* < 0.05, indicate a significant difference between the samples. The results show that the small-world properties in the delta and theta frequency bands show statistical differences in all tasks. On the other hand, only the network in the theta frequency band shows statistical differences in both small-world properties and global efficiency parameters. For the alpha band and beta band, statistical differences are only reflected in some tasks. The analysis results for the global efficiency show that the statistic F of the theta frequency band is much higher than that of other frequency bands in all tasks. The statistical results of the small-world attributes and global efficiency of all samples under different workload conditions are shown in Figure 11 and Figure 12.

Changes in small-world attribute values are related to the degree of aggregation of the overall network. When comparing the small-world attributes of functional brain networks in different frequency bands, it was observed that the structure of the network changes with the degree of the workload. In all three types of tasks, the small-world attribute values under the delta frequency band show a trend of first increasing and then decreasing, and the network small-world attribute values under high workloads are lower than those under low and medium workloads. In addition, the small-world attributes value of the theta frequency band is relatively high, and as the degree of cognitive workload increases, the small-world attributes value of the theta frequency band tends to decrease.

As the degree of cognitive workload increases, the global efficiency of the delta band and theta band gradually increases. The improvement in the global efficiency of the network indicates that the connectivity between nodes is enhanced, that is, the number of transit nodes required for interaction between nodes is reduced, the shortest path length between pairs of nodes is shorter, and the information transfer between various areas of the brain is more capable.

### 6.2. Network Local Parameter Analysis

In order to explore the changing rules of local parameters of the network in more detail, a single-factor variance analysis was conducted on the clustering coefficient and the local efficiency of the functional brain network nodes under each electrode node. At the same time, in order to further explore the characteristics of the functional brain network under different workload conditions, the number of nodes showing significant differences (*p* < 0.05) in the four frequency bands was counted. The proportion of the number of nodes with significant differences in each frequency band is shown in Table 5.

**Figure 13:**
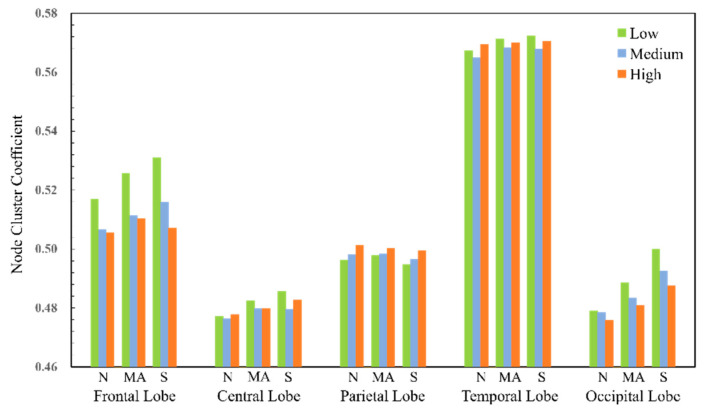
Node clustering coefficients of brain functional networks in theta band.

**Figure 14:**
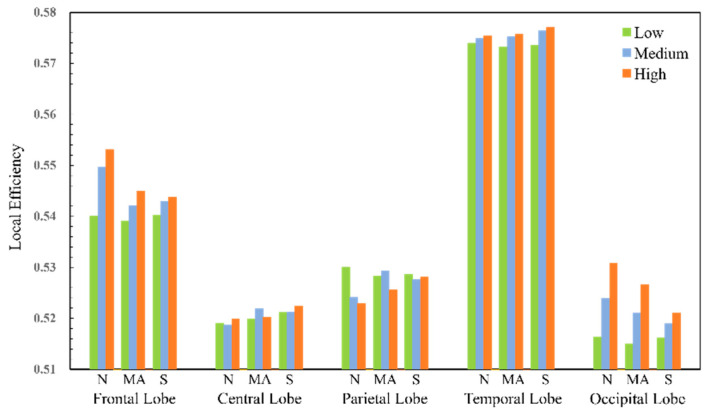
Local efficiency of functional brain networks in theta band.

For the four frequency bands, the number of nodes with significant differences in the theta frequency band is significantly higher than those in other frequency bands. Therefore, the local parameters of the nodes of the functional brain network in the theta frequency band will be analyzed in detail later. At the same time, the 59 electrode nodes are divided into five brain areas, the frontal lobe, central lobe, parietal lobe, occipital lobe, and temporal lobe, according to the distribution of each electrode. The local network parameters of each brain area are the average of the local attributes of the electrode nodes in that area, as shown in Figure 13 and Figure 14.

The higher the node clustering coefficient, the more likely the node is to be connected to its neighbor nodes. Under different workload levels, the clustering coefficient of the nodes in the temporal lobe part is higher, while that in the central lobe part is relatively low. On the one hand, as the cognitive workload increases, the electrode nodes in the frontal lobe become relatively large and show a stable downward trend, and the same downward trend is seen in the electrode nodes in the occipital lobe. On the other hand, the change trend of the node clustering coefficient of the electrode nodes in the parietal lobe is different from that of the frontal lobe and occipital lobe, showing an increasing state but a relatively low change amplitude. The local clustering coefficients of some electrode nodes in the temporal lobe under the medium workload were lower than those under the low workload and high workload conditions.

As the workload increases, the local efficiency of electrodes in the frontal lobe, parietal lobe, and temporal lobe shows an upward trend under different tasks. Among them, the local efficiency value of the electrodes in the temporal lobe is the highest, but its fluctuation range is small, while the frontal lobe also has a small fluctuation range. The changing trend of the electrode nodes in the occipital lobe region is more obvious. Changes in the local efficiency indicate that the pattern of information interaction between brain regions is changing, and as the cognitive workload gradually increases, some network nodes have faster information transfer between neighboring nodes.

### 6.3. Performance Comparison by Different Frequency Bands

**Figure 15:**
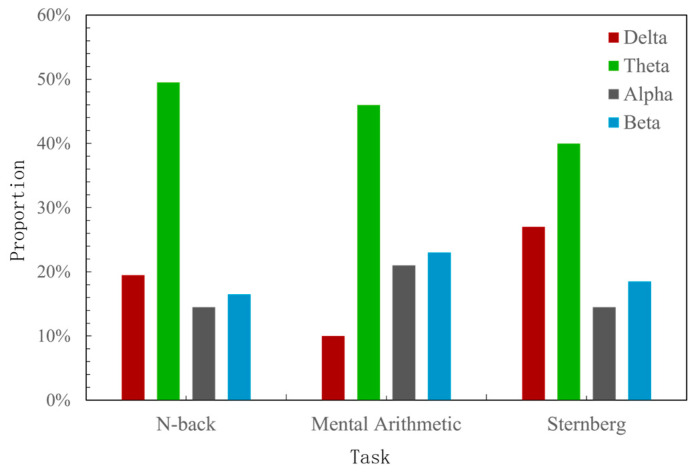
Statistics on the percentage of functional connections under each frequency band.

**Figure 16:**
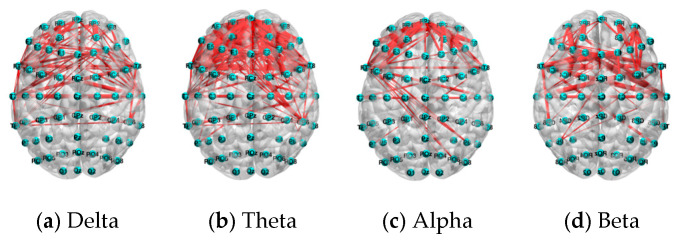
The distribution of the top 5% connections of PLV features under the N-back task.

**Figure 17:**
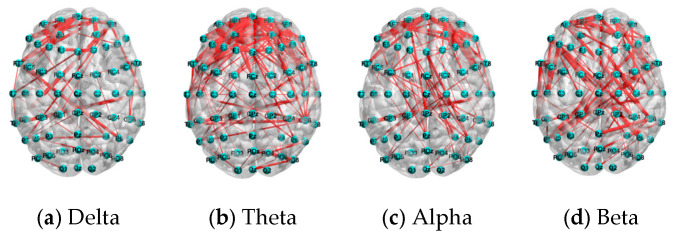
The distribution of the top 5% of connections of PLV features under the mental arithmetic task.

**Figure 18:**
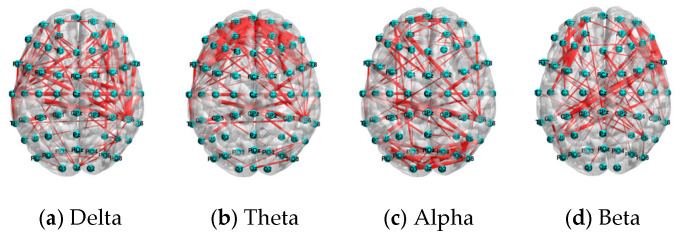
The distribution of the top 5% of connections of PLV features under the Sternberg task.

To analyze the EEG connectivity differences under different cognitive workload tasks, we used the PLV, sorted features by their contribution rate, and retained the top 5% of the 6844 features in the four bands, as shown in Figure 15. The theta band had the highest feature percentage in all tasks, especially in the N-back task (close to 50% of the total). Regarding the other bands, the beta band generally had more features than the alpha band, and the delta band’s feature percentage was unstable. Figure 16, Figure 17 and Figure 18 show the 5% most relevant features’ functional connectivities. The frontal lobe electrode node connections were significantly more numerous in this region than in the other regions. In the delta band, the N-back and mental arithmetic task connections were observed mainly in the frontal lobe, and the Sternberg task also had many right–temporal lobe connections. The theta band had the highest classification effect and the most high-correlation features, and its key connections were dense in the frontal lobe. In the alpha band, the features were more diverse. In the beta band, the occipital lobe connections were fewer, and these were observed mainly in the frontal and parietal lobes. These results show different brain functional connectivity activation areas in different tasks, but also some commonalities that need further exploration.

### 6.4. Cross-Task Classification Performance Based on COG-BCI Public Dataset via Subject-Dependent Mapping

To further validate the generalization ability of the model, we chose the public dataset COG-BCI for subject-dependent cross-task-based experiments and compared it with other studies, as shown in Table 6. The proposed model achieves 91.7% (binary), 86.5% (3-class), and 47.1% (4-class) classification accuracies on the task of multi-classification, significantly outperforming other methods. This shows that the proposed model has a good generalization ability to different datasets as well as classification forms.

## 7. Conclusions

In this paper, EEG signals under three cognitive workload paradigms with different levels were collected and used to identify the states of cognitive workload across subjects. The SGATCNs model is proposed, which aggregates spatial information of graph signal nodes in different frequency bands through the use of stacked graph attention layers, and deeply mines complementary information hidden between each frequency band through the use of convolution layers. According to the frequency domain features of EEG signals, the network structure in different frequency band is taken as a snapshot to construct discrete graph data. Functional brain network structures were constructed using different connectivity methods under different sparsities. Among them, the results of the cross-task cognitive workload recognition showed that the recognition accuracy of the network was the highest when the sparsity was 0.3, and the recognition accuracy of the functional brain network constructed based on PLV features was higher than that of other connectivity methods, reaching 65.11%. Moreover, the global parameters of the brain network in the theta band showed significance under different tasks, and the functional brain network in this band had the highest proportion of nodes with differences. In addition, the changes in the partial parameters were mainly concentrated in the frontal and occipital brain regions, and the node clustering coefficient and the local efficiency of the nodes in this part of the brain region showed stable downward and upward trends, respectively, and these changes were relatively large.

In the future, we will explore more diverse and complex real-world cognitive workload scenarios to further validate the model’s generalizability. Additionally, we will investigate the potential of combining the model with other techniques or modalities to improve the accuracy and reliability of cognitive workload assessment.

## Figures and Tables

**Table 1:** Classifier Parameter Settings.

	Classifier	Parameter
1	SGATCN	Epoch = 800, Learning rate: 0.001, batch_size = 30
2	GAT	Epoch = 500, Learning rate: 0.005, batch_size = 50
3	GCN	Epoch = 500, Learning rate: 0.01, batch_size = 50
4	SVM	C = 0.5
5	RF	n_estimators = 20, max_depth = 10
6	KNN	K = 15
7	LR	C = 0.5, max_iter = 1000

**Table 2:** Cognitive Workload Categorization Effects of Sternberg Tasks with Different Sparsity (%).

Sparsity		0.1	0.2	0.3	0.4	0.5	0.6	0.7	0.8	0.9
PLV	N	47.52	60.23	66.12	62.33	56.63	54.98	49.25	45.12	39.21
	MA	46.59	59.35	64.88	61.22	55.67	54.43	47.51	43.15	36.05
	S	46.92	57.86	64.35	60.54	53.21	52.21	48.35	44.12	37.15
PLI	N	44.53	57.98	59.62	54.31	53.71	46.83	45.01	41.68	36.9
	MA	43.64	57.44	58.84	54.11	52.72	47.53	44.52	41.96	36.68
	S	43.32	56.82	62.12	60.13	55.72	51.44	45.09	41.86	36.77
PCC	N	44.71	57.42	63.34	60.72	56.46	51.98	46.55	43.06	37.96
	MA	44.32	56.18	62.86	59.18	55.87	51.76	46.77	42.15	37.42
	S	43.55	56.23	58.76	53.12	52.17	46.07	44.18	40.28	35.74
MI	N	43.08	49.39	56.79	53.01	49.52	42.18	42.6	39.81	36.81
	MA	41.86	48.56	56.31	52.36	48.73	43.16	42.12	37.56	35.56
	S	41.17	48.1	55.83	51.22	48.66	43.62	41.08	37.91	35.91

**Table 3:** Task-Independent Cognitive Workload Recognition Results by Different Classifier (%).

	Classifier	Accuracy	Precision	F1 Scores
1	SGATCN	65.11	65.07	65.28
2	GCN	56.46	56.31	55.36
3	GAT	57.44	56.94	56.89
4	SVM	52.12	51.74	51.08
5	RF	48.85	49.82	47.73
6	KNN	45.89	46.26	45.45
7	LR	47.61	46.92	46.47

**Table 4:** Variance Analysis Results of Network Global Parameters (F(*p*)).

Network Global Parameters	Task	Delta	Theta	Alpha	Beta
Small-world properties	N	12.11 (<0.001)	20.727 (<0.001)	1.699 (0.182)	1.534 (<0.001)
	MA	6.31 (0.001)	9.994 (<0.001)	3.277 (0.037)	2.355 (0.094)
	S	4.28 (0.013)	8.477 (<0.001)	13.29 (<0.001)	7.078 (<0.001)
Global efficiency	N	11.354 (<0.001)	20.216 (<0.001)	0.782 (0.481)	0.985 (0.373)
	MA	2.951 (0.06)	38.731 (<0.001)	3.468 (0.031)	6.854 (<0.001)
	S	7.161 (<0.001)	14.596 (<0.001)	7.781 (<0.001)	8.851 (<0.001)

**Table 5:** Proportion of Nodes with Significant Differences in Local Parameters Under Each Frequency Band.

Feature	Task	Delta	Theta	Alpha	Beta
Node clustering coefficient	N	45.76%	57.63%	32.20%	38.98%
	MA	40.68%	54.24%	18.64%	35.59%
	S	28.81%	62.71%	40.68%	42.31%
local efficiency	N	42.37%	64.41%	27.12%	37.29%
	MA	32.20%	42.98%	16.95%	27.12%
	S	23.73%	57.63%	27.12%	35.59%

Note: N (N-back), MA (mental arithmetic), S (Sternberg).

**Table 6:** Comparative Experiments on Public Datasets.

Study	Classes	Accuracy
Zhou [18]	Binary	0.714
Baldwin [33]	Binary	0.448
Zhang [34]	Binary	0.889
Dimitrakopoulos [35]	Binary	0.870
Ke [36]	4-class	0.292
Guan [37]	3-class	0.813
Ours	Binary	0.917
	3-class	0.865
	4-class	0.471

## Data Availability

The data is unavailable due to privacy or ethical restrictions.
